# Evaluation of the Success Criteria for Zirconia Dental Implants: A Four-Year Clinical and Radiological Study

**DOI:** 10.1155/2013/463073

**Published:** 2013-08-26

**Authors:** Andrea Enrico Borgonovo, Rachele Censi, Virna Vavassori, Marcello Dolci, Josè Luis Calvo-Guirado, Rafael Arcesio Delgado Ruiz, Carlo Maiorana

**Affiliations:** ^1^Department of Implantology, Dental Clinic, Fondazione IRCCS Ca' Granda Ospedale Maggiore Policlinico, School of Oral Surgery, University of Milan, Milan, Italy; ^2^Department of Implantology and Periodontology III, Istituto Stomatologico Italiano, Milan, Italy; ^3^Faculty of Medicine and Dentistry, Murcia University, Spain

## Abstract

*Objectives*. The aim was to evaluate survival and success rates, soft tissue health, and radiographic marginal bone loss (MBL) of zirconia implants placed in the esthetic and posterior areas of the jaws and in association with multiple or single implant restorations after at least 6 months of definitive restoration. *Material and Methods*. 35 one-piece zirconium implants were utilized for single or partially edentulous ridges rehabilitation. All implants received immediate temporary restorations and six months after surgery were definitively restored. Every 6 months after implant placement, a clinical-radiographic evaluation was performed. For each radiograph, the measurements of MBL were calculated. *Results*. The results showed that the mean MBL at 48-month followup was 1.631 mm. The mean MBL during the first year of loading was not more significant for implants placed in the first molar regions than for those positioned in other areas. Moreover, no differences in marginal bone level changes were revealed for multiple and single implants, whereas MBL in the first year was observed to be slightly greater for implants placed in the maxilla than for those placed in the mandible. *Conclusion*. Zirconia showed a good marginal bone preservation that could be correlated with one-piece morphology and characteristics of zirconia implants.

## 1. Introduction

The use of endosseous implants achieves predictable results in terms of survival and success rates of oral rehabilitation [1]. More recently, greater interest is directed towards esthetics of the prosthetic rehabilitations. Successful esthetic results of dental implant placement require knowledge of essential biological concepts and skill in different surgical techniques [2]. 

Anyway, the surgical techniques or prosthetic [3] solutions by now proposed are not always sufficient to achieve long-term esthetic results; in fact, it was demonstrated that periimplant soft tissues tend to recede after positioning a definitive prosthetic restoration [4]. In order to avoid grayish transparency of titanium implants through soft tissues, ceramic materials were tested. All-ceramic dental implants were introduced in dental implantology as an alternative to titanium implants. Another reason to find an alternative material to titanium was sensibilization, possible release of metallic ions, and allergy to this material, as reported in some studies [5, 6]. Alumina was one of the first ceramic materials used but because of its hardness combined to a low flectural and fracture strength poor long-term results were achieved and this material was abandoned [7]. 

More recently, research was oriented towards new generation ceramic materials such as zirconia, which has more favorable mechanical properties (high flexural strength 900–1200 Mpa, hardness 1200 Wickers, and Weibull modulus 10–12). In addition, zirconia has a high biocompatibility and low plaque adhesion [8, 9] and several animal studies showed long-term osseointegration of zirconia dental implants and a bone-to-implant contact similar to titanium [10–13]. Due to its favorable aesthetic characteristics, zirconia can be successfully used in case of thin biotypes or soft tissue recessions. 

Moreover, studies [4] demonstrate that perimplant soft tissues tend to recede after positioning a definitive prosthetic restoration. 

The purposes of this study were to evaluate survival and success criteria of endosseous one-piece yttrium-stabilized zirconia dental implants during a follow-up period of 12–48 months after insertion, appreciate periimplant soft tissue health, and consider periimplant marginal bone remodeling.

## 2. Materials and Methods

### 2.1. Implant System

**Figure 1:**
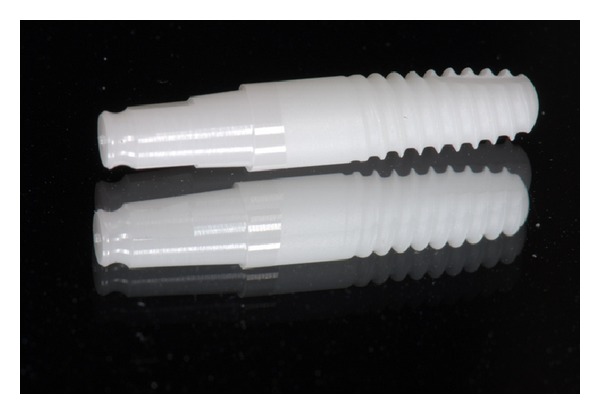
Implant characteristics.

In this study, WhiteSky dental implant (Bredent, Senden, Germany) was used. WhiteSky is a one-piece implant characterized by a conical body with double threads and rounded apex and is made of sintered and yttrium-stabilized zirconium oxide. The endosseous implant surface is treated with a sandblasting process that determines microscopical surface characteristics of medium rugosity (*R*_*a*_ = 0.9-1 m) similar to those of last generation titanium implants, whereas in the gingival area the implant features a machined neck with a height of 2 mm. The abutment surface is also machined and has a length of 6.8 mm (Figure 1).

### 2.2. Patients Selection

For this study, patients were selected according to the following criteria.

All implant sites should present adequate bone volume (height > 8 mm, thickness > 4 mm).

Absence of systemic contraindications (metabolic disorders, immunodeficiency, hemathological diseases, neoplastic diseases, bisphosphonates history, and smoking > 10 cigarettes/day) is evident.

Absence of local contraindications (head and neck radiotherapy, poor oral hygiene, active periodontal disease, and parafunctions) is evident.

Exclusion criteria were determined by the presence of one or more of the systemic or local contraindications plus patients aged <18 years, patients with total edentulism, patients with detected bruxism and regenerative procedures previous to implant insertion.

Starting from January 2007, and recruited in a period of one year, 13 patients were included in the study. The data were recorded to July 2012 when the implants had a minimal observation period of 4 years. 

Average age was 60 years (range from 38 to 75). Twelve patients were men and one was woman. 

35 one-piece endosseous dental implants made of sinterized and yttrium-stabilized zirconium oxide were used for the rehabilitation of single tooth or partially edentulous ridge. 

Three patients, with seven implants placed, were dropped out the protocol because they did not attend to followup. 

### 2.3. Presurgical Protocol

Prior to surgery patients had to:

- take orthopantomograph or standardized endoral radiographic,
- take Ct san,
- undergo professional oral hygiene (7 days before surgery),
- start mouth rinsing twice a day with chlorhexidine 0.2% (Corsodyl, Glaxo, UK) 2-3 days before surgery and continue for 2 weeks after surgery,
- take antibiotic prophylaxis with amoxicillin + clavulanic acid (Laboratori Eurogenerici, Milano, Italy) 2 g 1 hour prior to surgery.

### 2.4. Surgical Protocol

All patients were previously informed about zirconia implants and possible alternatives and gave a written consent.

Implants were positioned according to the guide of a surgical mask obtained by a diagnostic wax-up. After reflecting a full thickness flap, implant site preparation was performed in order to leave implant abutments with machined neck to heal transmucosally, whereas implant body rough surface was left completely inside the bone. All implants were inserted using both a surgical motor and manually. In case of fenestrations or dehiscences, regenerative procedures by resorbable membranes (Bio-Gide, Geistlich Pharma, AG Wolhusen, Switzerland) and bone substitutes (Bio-Oss, Geistlich Pharma, AG Wolhusen, Switzerland) were performed. The implant stability was evaluated with the torque at insertion. Flaps were sutured with 4/0 monofilament suture (Premilene,Braun, Melsungen, Germany). When necessary, flaps were released through periosteal incisions in order to attain a primary wound closure. Patients were given oral hygiene suggestions and were instructed not to chew or eat on implant site until healing was completed. Antibiotic therapy and chlorhexidine mouth rinses were continued for 7 days and Paracetamol 500 mg (Tachipirina, Angelini, Roma, Italy) was prescribed and adopted by the patients when needed. Sutures were removed 7 days after surgery. Follow-up controls were programmed after 1 week, 2 weeks, and subsequently once a month for the following 6 months.

### 2.5. Prosthetic Protocol

Immediately after surgery, implant abutments were prepared in order to correct axis and length using double diamond burs suited for zirconia (Eterna,Bredent, Senden, Germany), and water cooling and temporary restorations were relined with acrylic resin. Single implants were cemented avoiding centric and eccentric contacts and stabilized to the adjacent teeth using composite wings for at least 6–8 weeks in order to reduce the risk of failure due to micromovements. Multiple implants were connected together by a provisional restoration and when possible excluded from occlusal contacs.

Six months after surgery, implants were finally restored by all ceramic zirconia crowns or bridges made with CAD-CAM system (LAVA, 3M ESPE, St. Paul, MN) and cemented with a glass ionomer cement (GC Fuji, CEM GC America, Alsip, IL).

### 2.6. Follow-Up Protocol

The protocol included clinical-radiographic examinations every 6 months after surgery as follows.

Clinical evaluation (presence or absence of mobility, self-reported pain, or paresthesia and assessment of the integrity of the final prosthesis) is evident.

Periodontal evaluation with calibrated probe (Hu-Friedy, N. Rockwell, Chicago, IL) in order to evaluate probing pocket depth (PPD), plaque index (PI), and bleeding on probing (BOP) is evident.

Survival criteria were identified as the survival of loaded functionalized asymptomatic implants.

Success criteria were formulated according to the following parameters:

1. mobility is not present,
2. self-reported pain or paresthesia is not present,
3. periimplant radiolucency is not present,
4. periimplant marginal bone loss inferior to 1.5 mm during the first year in function and an annual bone loss thereafter are not exceeding 0.2 mm.

### 2.7. Radiographic Evaluation

**Figure 2:**
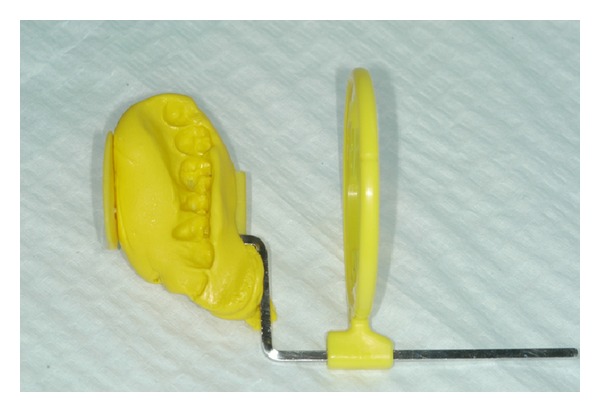
Standardized periapical radiographs were obtained using the Rinn alignment system with customized silicone bite.

**Figure 3:**
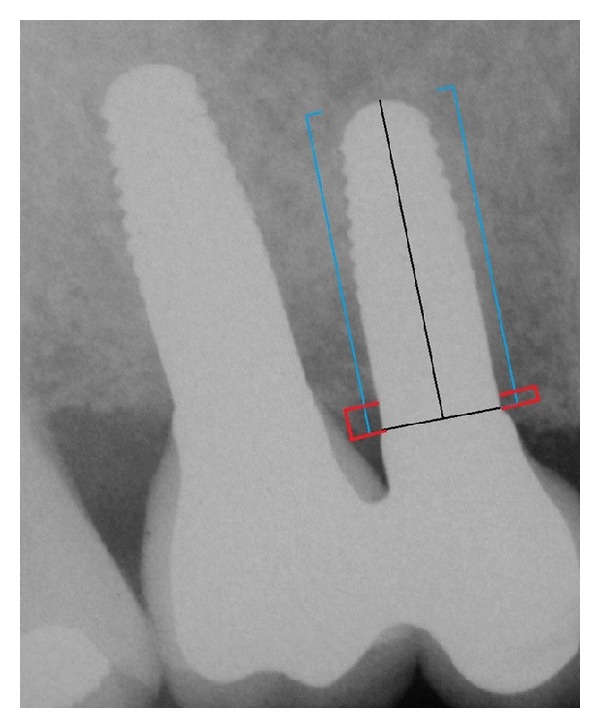
X-ray picture 12 months after surgery. The blue lines indicate the implant length; the red lines indicate the periimplant bone levels.

According to this protocol, standardized periapical radiographs should be taken at the time of implant placement and 6 months, 12 months, 24 months, 36 months, and 48 months after, using a customized bite record made with Orthogum (Zhermack, Badia Polesine, Rovigo, Italy) on a rinn XCO Ring positioner (Dentsply, Constanz, Germany) (Figure 2). Radiographs were acquired and converted into digital images with a scanner (Epson 1680 Pro, Seiko Epson Cooperation, Nagano, Japan) and saved in  .JPG format. Each image was processed with a specific software (CorelDraw 10.0, Corel Corp and Coral Ltd, Ottawa, Canada) and analyzed at ×20 magnification in order to calculate marginal bone loss. Mesial and distal marginal bone levels of all the implants were determined at baseline and recall evaluations. The known length of the implant (measured from the implant shoulder to the implant apex) according to the manufacturer was used as reference point. The distance from implant shoulder to crestal bone level was measured on the magnified images (Figure 3). To account for variability, the implant dimension (length) on the magnified X-ray was measured and compared to the real dimension, and ratios were calculated to adjust for distortion. Bone levels changes were calculated at the distal and mesial surfaces of all implants by applying the distortion coefficient. 

### 2.8. Statistical Evaluation

Data analysis was performed with descriptive statistics. Mean and standard deviation values were recorded and the paired Student's *t*-test was used for comparison of mean values between groups (multiple-single implants; implants placed in maxilla or mandible; implants placed in esthetic or posterior regions of the jaws). Confidence interval was set at 99% mean for all measurements. Statistically significant differences were set at *P* = .01. 

## 3. Results

The data reported in this study refer exclusively to 28 implants. 

Mean implant diameter was 4 mm and mean implant length was 12.3 ± 1.28 mm. The range of length for the implants considered in this study was from 10 to 14 mm.

20 implants were placed in the maxilla, whereas 8 implants were inserted in the mandible.

Six implants replaced missing first molars both in mandible and maxilla, and the remaining implants were placed in other regions (from 1.5 to 2.5 and from 3.5 to 4.5). 20 implants were used for multiple teeth replacement.

All implants were immediately restored with temporary acrylic resin crowns or bridges (multiple implants were splinted together by provisional restoration), and definitive restorations were positioned after a healing time of 6 months. The implants were finally restored by all ceramic zirconia crowns or bridges made with CAD-CAM system, as described.

During the 48 months of followup, no implant failure was reported, no pain, and no paresthesia, and at the radiographic evaluation periimplant radiolucency was present in none of the implants. 

Survival and success rates within follow-up period were therefore 100% (Table 1). 

Every 6 months after the final prosthetic rehabilitation, periodontal indexes were registered for each patient at each implant site (plaque index, bleeding on probing, probing pocket depth, and implant mobility), and standardized radiographs were taken using the long-cone paralleling technique. The periodontal indexes are reported in Table 2.

Radiographic evaluation indicated that mean marginal bone loss was 1.38 ± 0.02 mm 6 months after implant insertion; 0.41 ± 0.05 mm 6 months after prosthetic finalization except for 2 sites where it resulted as 1.5 ± 0.06 mm. A minimal bone remodelling with a further marginal bone loss of −0.23 mm, 0.021 mm at 36 months, and 0.05 at 48-month followup was observed. The mean marginal bone loss at 48 months of followup was 1.631 mm. 

**Figure 4:**
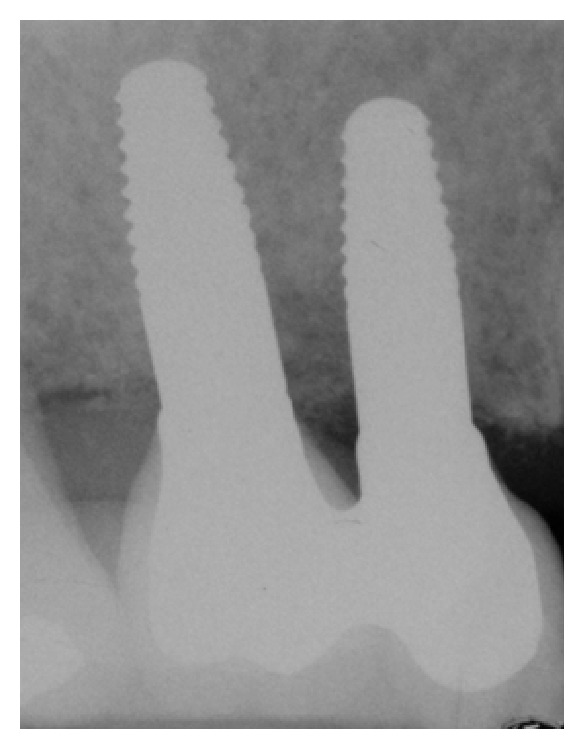
X-ray picture 24 months after surgery.

**Figure 5:**
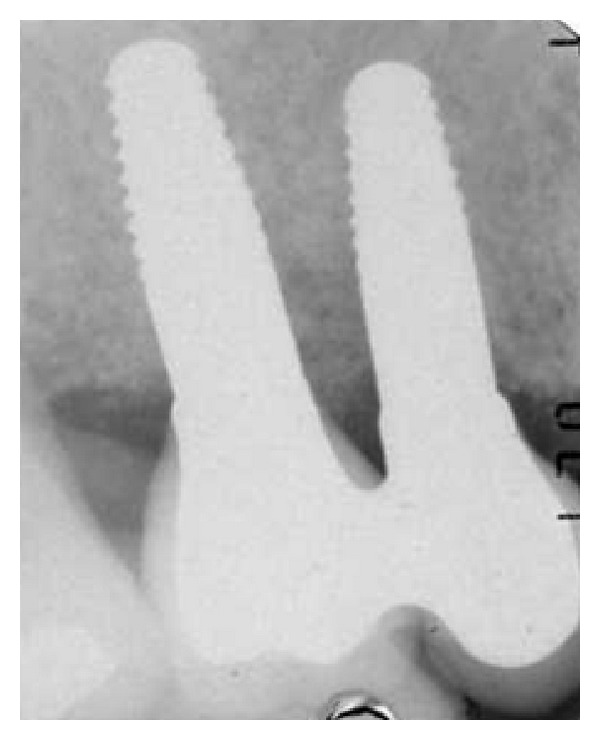
X-ray picture 48 months after surgery. No differences in marginal bone level changes can be observed from 12 to 48 months after surgery.

For implants placed in the maxilla, the average marginal bone loss from baseline to 6 months was 1.37 ± 0.27 mm; from 6 to 12 months was 0.677 ± 0.7 mm; from 12 to 24 months was −0.078 ± 0.51 mm; from 24 to 36 months was 0.021 ± 0.39 mm (Figures 4 and 5). For implants placed in the mandible, the radiographically determined mean MBL amounted to 0.59 ± 0.73 mm during the first year of loading while no MBL change data were reported after first year in function. 

The marginal bone loss during the first year of loading was more significant for implants placed in maxilla than for those positioned in the mandible (*P* < .019).

The MBL changes from prosthetic restoration to 1 year of loading were 0.445 ± 0.87 mm for implants placed in the first molar region and 0.4 ± 0.7 mm for implants placed in other regions. After 4 years of function, the MBL change increased, respectively, by 0.02 mm and 0.169 mm. The difference between the two groups at 4 years was not statistically significant.

It was investigated that from baseline to 48 months after surgery, the mean bone resorption values were +1.2081 mm for multiple implants and +1.2088 mm for single implants. The difference between the two groups was not statistically significant.

## 4. Discussion

Several studies in animal models showed successful osseointegration of zirconia dental implants under both unloaded and loaded conditions and bone-to-implant contact values similar to those of titanium. Scarano et al. [8] investigated the bone response to 20 YTZP implants inserted in the tibiae of five rabbits. According to the authors, all implants were osseointegrated without signs of inflammation or mobility. The mean BIC was calculated to be 68%. 

Absence of signs of marginal bone loss around implants surface indicates maintained integration between the implant fixture and the surrounding bone. However, the finding of periimplant bone remodelling must be carefully considered because the marginal bone loss which may be detected around implants after beginning of function should be distinguished from the bone loss that is affected by one or more of the following factors: (1) traumatic surgical technique [14]; (2) excessive loading conditions [15]; (3) location, shape, and size of the implant abutment microgap and its microbial contamination [16]; (4) biologic width and soft tissue considerations [17]; (5) periimplant inflammatory infiltrate [18]; (6) implant and prosthetic components micromovements [19]; (7) repeated screwing and unscrewing [20]; (8) implant-neck geometry [21]; and (9) infectious process [22].

According to several studies investigating criteria for implant treatment success [23, 24], a marginal bone loss of 1.5 mm during the first year in function and an annual bone loss not exceeding 0.2 mm thereafter is considered acceptable. Brägger et al. [25] defined a radiographic criterion for implant success, a perimplant bone resorption below the limits of 0.9 to 1.6 mm during the first year in function. 

The results of this study showed a mean marginal bone loss of 1.38 mm 6 months after implant insertion; 0.41 mm from 6 to 12 months, −0.23 mm from 12 to 24 months, 0.021 mm from 24 to 36 months; 0.05 mm from 36 to 48 months. The mean marginal bone loss at 48 months of followup was 1.631 mm.

In the study, it was observed that marginal bone loss in the first year is slightly greater for implants placed in the maxilla than for those placed in the mandible. These data could be explained by the fact that there are differences in the remodelling capacity and rate between maxillary and mandibular bone also due to the different bone quality and density in the different sites. 

Periimplant bone level changes during the first year of loading were not more significant for implants placed in the first molar regions than for other areas. The variation from final prosthetic restoration up to 1 year of loading was 0.445 ± 0.87 mm for implant placed in the first molar region and 0.4 ± 0.7 mm for implants placed in other regions. After 4 years of function, the MBL change had increased, respectively, to 0.02 mm and 0.169 mm. 

No differences in marginal bone level changes were observed between multiple and single implants since baseline to 4 years after surgery; the mean bone resorption values were 1.2081 mm for multiple implants and 1.2088 mm for single implants.

Greater bone loss occurs during the first year of function, and it is related to maturation of bone after the surgery and adaption of bone to withstand functional forces. In this study, the values of marginal bone loss were within the limits of 0.9 to 1.6 mm, considered to be acceptable for the first year of loading. From 12 to 24 months after implant positioning, an improvement of MBL values was observed probably due to the formation of new bone trabeculae as a result of maturation of bone. After the second year of functioning, the annual amount of bone loss was <0.2 mm. 

A large number of studies evaluated the causes of early marginal bone loss around implants. Abrahamsson and Berglundh [26] considered the effects of different implant surfaces on marginal bone levels alteration and did not observe any evidence of improved marginal bone preservation for any particular implant surface and configuration modification. 

However, one-piece morphology of zirconia dental implants can influence marginal bone loss. In fact, it has been proposed that periimplant marginal bone loss is more extended around two-piece implants than around one-piece implants as a result of the location of the microgap [27, 28]. The presence of the microgap leads to bacterial leakage and a microbial colonization of the gap at the bone level. Periimplant soft tissues develop an inflammatory response which promotes osteoclast formation and activation to result in alveolar bone loss. Bone remodeling will progress until the biologic width has been created and stabilized. Not only does this width progress apically, along the vertical axis, but according to studies conducted by Tarnow et al. [29] there is also a horizontal component amounting to 1–1.5 mm.

Furthermore, it is important to consider that plaque accumulation on implant or abutment surface induces a gingival inflammatory reaction [30] and consequently a progressive bone loss [31]. In particular, roughness plays an important role in the bacterial adhesion and this relationship has been demonstrated in several in vivo and in vitro studies [32, 33]. Scarano et al. [34] showed that zirconium oxide presented a significant reduction of the presence of bacteria, and Rimondini et al. [35] found that yttrium-stabilized tetragonal zirconia surfaces accumulated significantly fewer bacteria than titanium. 

The reduced bacterial adhesion on zirconia implants surface promotes early formation of the biologic width and therefore the formation of a mucosal seal that stops early marginal bone resorption [36–40].

## 5. Conclusions

Between the limits of this human clinical study, the following could be stated.

1. The crestal bone level of zirconia dental implants suffers a slight reduction of 1.5 mm after 4 years followup and according to several studies, when using a radiographic criterion for implant success, marginal bone loss below 0.9–1.6 mm during the first year of function can be considered acceptable. This periimplant bone preservation may be associated with the absence of microgap between fixture and abutment since zirconia dental implants are one-piece implants. Moreover, zirconia is characterized by a high biocompatibility and it accumulates significantly fewer bacteria than titanium.
2. No bleeding, minimal plaque index of 0.5, and probing depth of 3.19 mm could be expected in zirconia dental implants.
3. The absence of mobility with the previous parameters is the key of success criteria of zirconia dental implants; due to these characteristics zirconia implants may be considered as reliable as titanium in terms of osseointegration and biological tissues response.

The data reported in this study even if limited are encouraging. For this reason, further long-term clinical studies regarding the success rates and clinical outcome of zirconia dental implants are needed.

## Figures and Tables

**Table 1:** Survival and success rates.

	6 months	12 months	18 months	24 months	36 months	48 months
Success criteria						
Mobility	−	−	−	−	−	−
Paresthesia	−	−	−	−	−	−
Radiolucency	−	−	−	−	−	−
MBL < 1.5 mm (1° year) + <0.2 mm (following years)	+	+	+	+	+	+
Survival criteria						
Loaded functionalized asynthomatic implants	+	+	+	+	+	+

(+) present/(−) absent.

**Table 2:** Periodontal indexes.

	Months after implant insertion					
Periodontal Indexes	6 months	12 months	18 months	24 months	36 months	48 months
PI	1	0.5	0.75	0.5	0.43	0
BOP	1	0.25	0.5	0.5	0.86	0.15
PPD (mm)						
MV	4 (±0.7)	3 (±0.8)	3.5 (±1.3)	2.75 (±0.5)	3.9 (±1.28)	3.9 (±0.7)
V	2.5 (±0.7)	2.75 (±0.5)	3 (±0.8)	2.25 (±0.9)	2.7 (±0.8)	2.43 (±0.5)
DV	3.5	2.75 (±0.5)	3.5 (±1)	2.25 (±0.5)	3.3 (±0.9)	2.71 (±0.5)
MP	3	3.25 (±1.25)	4 (±0.8)	3.5 (±1.3)	3.4 (±0.8)	3.9 (±0.7)
P	2	3	2.25 (±0.9)	2.5 (±1.3)	2.9 (±1.1)	2.6 (±0.8)
DP	3	2.75 (±0.5)	3 (±0.8)	3.25 (±1.3)	3.5 (±1.2)	3.6 (±0.8)
PPD (mean)	**3.0**	**2.917**	**3.208**	**2.75**	** 3.283 **	** 3.19**
